# Less Invasive Surfactant Administration Compared With Intubation-Surfactant-Extubation (INSURE), Nebulised Surfactant, and Continuous Positive Airway Pressure (CPAP) Alone in Preterm Infants With Respiratory Distress Syndrome: A Systematic Review

**DOI:** 10.7759/cureus.116702

**Published:** 2026-09-22

**Authors:** Nashwa Noureldeen Abdelgadir, Rasha Omer Babiker Mohamed, Saja Dafa Alla Bakhit Osman, Safaa Bushra Mohieldin Sultan, Esra Abdallah Ahmed Mohamed, Alaa Mohamed Elamin Mobark Osman, Reem Ismail Elfadol Biuo

**Affiliations:** 1 Pediatrics, Aliaa Specialist Hospital, Gezira, SDN; 2 Trauma and Orthopedics, Nobles Hospital, Douglas, IMN; 3 Pediatrics, Burjeel Royal Hospital, Al Ain, ARE; 4 Pediatrics, Care Medical Hospital - Al Rawabi, Riyadh, SAU; 5 College of Medicine, University of Khartoum, Khartoum, SDN; 6 Pathology, Assiut University, Assiut, EGY; 7 Pediatrics and Child Health, Doctor Mohammed Faqih Hospital, Riyadh, SAU

**Keywords:** continuous positive airway pressure, insure, less invasive surfactant administration, minimally invasive surfactant therapy, nebulised surfactant, preterm infant, respiratory distress syndrome

## Abstract

The optimal route of surfactant delivery in preterm infants, supported non-invasively, remains contested. Less invasive surfactant administration (LISA), or minimally invasive surfactant therapy (MIST), has been compared with intubation-surfactant-extubation (INSURE), with nebulised surfactant, and with continuous positive airway pressure (CPAP) alone, with inconsistent results.

Five databases and registers were searched from January 2021 to July 2026 for randomised controlled trials (RCTs) in preterm infants (<37 weeks) with respiratory distress syndrome. The review was not prospectively registered. Two reviewers independently screened records, extracted data, and applied the revised Cochrane risk-of-bias tool (RoB 2). Reporting followed Preferred Reporting Items for Systematic Reviews and Meta-Analyses (PRISMA) 2020 and, because no meta-analysis was performed, the Synthesis Without Meta-analysis guideline; certainty was rated using the Grading of Recommendations Assessment, Development and Evaluation (GRADE).

Eleven RCTs (1,455 infants) were included: eight compared LISA or MIST with INSURE (n = 792), one compared MIST with sham treatment and CPAP alone (n = 485), one compared nebulised poractant alfa with CPAP alone (n = 129), and one compared nebulised surfactant with LISA (n = 49). Compared with INSURE, three trials reported reduced invasive ventilation, but two of these were at high risk of bias, while all five trials showing no difference were at low risk or raised only some concerns; risk of bias and background ventilation mode are therefore confounded, and certainty was very low. Compared with CPAP alone, MIST halved intubation within 72 hours (36.5% compared with 72.1%; risk ratio: 0.50, 95% CI: 0.40-0.64; high certainty) and reduced pneumothorax. In that trial, bronchopulmonary dysplasia among survivors was reduced (0.83, 0.70-0.98), whereas the separate composite of death or BPD was not (0.87, 0.74-1.03). Nebulised surfactant did not reduce respiratory failure compared with CPAP alone.

LISA reduces early invasive ventilation compared with CPAP alone. Evidence against INSURE is heterogeneous and of very low certainty, and any attenuation with nasal intermittent positive pressure ventilation is confounded by trial quality and requires prospective testing. Nebulised surfactant remains insufficiently supported, and long-term outcomes are uncertain.

## Introduction and background

Respiratory distress syndrome (RDS) arising from developmental deficiency of pulmonary surfactant remains the single most frequent respiratory diagnosis of the preterm infant and a principal determinant of neonatal mortality, chronic lung disease, and prolonged intensive care utilisation. Its incidence is inversely related to gestational age. The most recent national analysis of United States inpatient data available to us, published in 2019, showed that RDS continued to affect a substantial and, in some strata, increasing proportion of preterm admissions despite the widespread availability of antenatal corticosteroids and exogenous surfactant [1,2]. Exogenous surfactant replacement, first shown to improve survival when combined with nasal continuous positive airway pressure (CPAP) three decades ago, corrects alveolar instability, restores functional residual capacity, and reduces the work of breathing [3]. European consensus guidance now recommends that spontaneously breathing preterm infants be stabilised on CPAP from birth, with rescue surfactant administered when the fraction of inspired oxygen (FiO2) requirement indicates evolving RDS [1]. The threshold at which rescue surfactant should be triggered is not settled: values between 0.30 and 0.60 have been used across major trials and continue to differ between units and guidelines. The therapeutic problem that follows from a CPAP-first strategy is mechanical rather than pharmacological because an infant maintained on CPAP has no endotracheal conduit through which surfactant can conventionally be instilled.

Two broad solutions have been developed. The intubation-surfactant-extubation (INSURE) technique, introduced to limit the duration of positive pressure ventilation, involves brief laryngoscopy and tracheal intubation, bolus surfactant instillation, and rapid extubation back to non-invasive support [3]. Although INSURE reduces exposure to sustained mechanical ventilation, it still requires airway instrumentation and delivers surfactant during a short period of positive pressure inflation that may itself contribute to volutrauma, and a proportion of infants fail extubation and remain ventilated. INSURE is often accompanied by sedative premedication, although practice varies substantially between centres and national guidelines, and some units perform the procedure awake. Failure of CPAP is not a benign event: large cohort analyses have shown that infants who fail CPAP and require delayed intubation experience substantially higher rates of death or bronchopulmonary dysplasia (BPD) than those supported successfully non-invasively [4]. Less invasive surfactant administration (LISA), also described as minimally invasive surfactant therapy (MIST) or surfactant administration via thin catheter, was developed to resolve this tension by instilling surfactant through a fine catheter passed briefly between the vocal cords while the infant continues to breathe spontaneously [5]. Aerosolised or nebulised surfactant represents a third, entirely non-instrumental approach in which surfactant is delivered in-line with the non-invasive circuit, obviating laryngoscopy altogether.

The non-invasive support on which these techniques are superimposed is itself not uniform, and the distinction matters for what follows. Nasal CPAP delivers a single continuous distending pressure throughout the respiratory cycle. Nasal intermittent positive pressure ventilation (NIPPV) superimposes intermittent peak inflations on that continuous pressure and therefore delivers a higher mean airway pressure, may augment tidal volume and minute ventilation, and reduces the frequency of extubation failure in some populations. Two units applying an identical surfactant technique may therefore be providing materially different background respiratory support, a source of heterogeneity that has received little attention. The outcome against which these strategies are conventionally judged is the composite of death or BPD at 36 weeks' postmenstrual age, used because death and chronic lung disease are competing outcomes that cannot be interpreted separately and because 36 weeks is the point at which continuing oxygen or pressure dependence best predicts later respiratory morbidity. BPD is multifactorial, arising from antenatal inflammation, postnatal ventilation, oxygen exposure, infection, and nutrition, so a technique can plausibly halve the need for early intubation without shifting this composite at all.

Evidence supporting LISA over endotracheal delivery has accumulated rapidly. A Cochrane review of surfactant therapy via thin catheter reported reductions in the composite of death or BPD, in the need for mechanical ventilation, and in severe intraventricular haemorrhage [5]; a network meta-analysis of surfactant delivery strategies ranked thin-catheter administration ahead of INSURE for mortality and major respiratory morbidity [6]; and large registry data from extremely preterm infants below 27 weeks' gestation have associated less invasive delivery with improved survival without major morbidity [7]. What none of these syntheses has examined is whether the mode of non-invasive support used as background therapy modifies the treatment effect, and each necessarily predates the most recent generation of trials, several of which come from low- and middle-income settings and use NIPPV rather than CPAP. The technique also remains operator-dependent, is associated with laryngoscopy-related bradycardia, desaturation, and surfactant reflux, and has attracted explicit calls for methodological caution regarding catheter design, dosing, sedation, and the generalisability of trials conducted in units with high procedural expertise [8]. This review was therefore undertaken to assemble the randomised trials published between 2021 and 2026 that compare LISA with INSURE, with nebulised surfactant, or with CPAP alone, to appraise them with the risk-of-bias tool (RoB 2) and Grading of Recommendations Assessment, Development and Evaluation (GRADE), and to describe their findings on respiratory, mortality, morbidity, and procedural safety outcomes. We state at the outset that the question of effect modification by background ventilation mode was not prespecified: it arose during the synthesis, when the pattern of null results became apparent, and it is presented throughout as a hypothesis generated by these data rather than as an established effect.

## Review

Methodology

Protocol and Registration

No review protocol was prepared in advance, and the review was not prospectively registered in PROSPERO or any equivalent register. We state this explicitly because compliance with reporting guidelines does not establish that methods were prespecified, and because two decisions described below, the publication window and the examination of background ventilation mode, were taken after the initial search rather than before it. Readers should weight those two elements accordingly. The review is reported in accordance with the Preferred Reporting Items for Systematic Reviews and Meta-Analyses (PRISMA) 2020 statement [9]. No ethical approval or patient consent was required because only previously published aggregate data were used.

Eligibility Criteria

Eligibility was defined using the Population, Intervention, Comparator, Outcomes, and Study design (PICOS) framework, summarised in Table 1. Randomised controlled trials (RCTs) were eligible if they enrolled preterm infants born before 37 weeks' gestation with established or evolving RDS who were breathing spontaneously on non-invasive support and met criteria for exogenous surfactant; if the intervention was LISA (or equivalently MIST, surfactant without endotracheal tube intubation (SurE), or non-intubated surfactant administration (NISA)) or nebulised surfactant delivered in-line with non-invasive support; and if the comparator was INSURE, nebulised surfactant, or continuation of CPAP alone, including sham-controlled designs. Reviews, meta-analyses, conference abstracts, editorials, letters, case reports, protocols, pre-prints, and non-randomised studies were excluded, as were trials comparing LISA with sustained endotracheal ventilation, comparing catheters or surfactant preparations using an identical technique, or comparing modes of non-invasive support alone.

**Table 1:** Eligibility criteria (PICOS framework) BPD: bronchopulmonary dysplasia; CPAP: continuous positive airway pressure; INSURE: intubation-surfactant-extubation; IVH: intraventricular haemorrhage; LISA: less invasive surfactant administration; MIST: minimally invasive surfactant therapy; NEC: necrotising enterocolitis; NIPPV: nasal intermittent positive pressure ventilation; NISA: non-intubated surfactant administration; PDA: patent ductus arteriosus; PICOS: Population, Intervention, Comparator, Outcomes, Study design; PMA: postmenstrual age; RDS: respiratory distress syndrome; ROP: retinopathy of prematurity; SurE: surfactant without endotracheal tube intubation.

Domain	Included	Excluded
Population	Preterm infants <37 weeks' gestation with established or evolving RDS; spontaneously breathing on non-invasive support; meeting criteria for exogenous surfactant	Term infants; intubation before enrolment; respiratory failure from another cause; major congenital anomalies or absent respiratory effort
Intervention	LISA via thin catheter during spontaneous breathing (equivalently MIST, SurE, NISA, Hobart method); or nebulised surfactant in-line with non-invasive support	Endotracheal surfactant with sustained ventilation; laryngeal mask, supraglottic or pharyngeal delivery; prophylactic delivery-room surfactant; drug combinations
Comparator	INSURE; nebulised surfactant; or continuation of CPAP alone, including sham-controlled designs	Comparisons of two catheters, surfactant preparations or doses using an identical technique; CPAP compared with NIPPV alone; lung recruitment before surfactant
Outcomes	Primary: intubation or invasive ventilation within 72 h. Secondary: death or BPD at 36 weeks PMA (reported separately from BPD among survivors), mortality, severe IVH, air leak, PDA, NEC, ROP, repeat dose, durations of support and stay, procedural adverse events	Studies reporting only physiological, biochemical, imaging, in-vitro, cost, or feasibility endpoints; outcomes not separable by randomised allocation
Study design	Randomised controlled trials published as full peer-reviewed articles, 1 January 2021 to 31 July 2026 (a pragmatic window applied after the initial search; see Methods)	Reviews, meta-analyses, abstracts, editorials, letters, case series, protocols, pre-prints, non-randomised and animal studies; publications before 2021

Information Sources, Search Strategy, and Publication Window

PubMed, Web of Science, Scopus, Embase, and ClinicalTrials.gov were searched from 1 January 2021 to 31 July 2026; each source was last searched on 31 July 2026. The search combined controlled vocabulary (MeSH and Emtree) with free-text terms across three concept blocks: population, intervention, and study design. A representative PubMed strategy was: ("infant, premature"[MeSH] OR preterm* OR premature OR neonate* OR newborn*) AND ("respiratory distress syndrome, newborn"[MeSH] OR "respiratory distress syndrome" OR RDS OR "hyaline membrane disease") AND ("pulmonary surfactants"[MeSH] OR surfactant* OR poractant OR beractant OR calfactant OR "bovine lipid extract") AND (LISA OR "less invasive surfactant" OR "minimally invasive surfactant" OR MIST OR "thin catheter" OR SurE OR NISA OR INSURE OR "intubation surfactant extubation" OR nebulis* OR nebuliz* OR aerosol* OR "vibrating mesh" OR "continuous positive airway pressure" OR CPAP) AND (randomized[pt] OR randomised OR "controlled trial" OR trial). Reference lists of all included trials and of recent syntheses on surfactant delivery were hand-searched, and forward citation tracking was performed in Google Scholar. Records retrieved from ClinicalTrials.gov were handled alongside database records in a single screening stream; registered trials that were ongoing or had no full peer-reviewed report were not eligible and are considered under knowledge gaps.

The Cochrane Central Register of Controlled Trials (CENTRAL), CINAHL, and regional indices such as IMSEAR and IndMED were not searched. Because CENTRAL is the standard source for randomised trials and because most included trials originate in South Asia, the Middle East, and North Africa, this is a material limitation and is acknowledged as such.

The restriction to publications from 1 January 2021 onwards was not prespecified. It was applied pragmatically after the initial search, when it became clear that more than 10 eligible trials had been retrieved, in order to confine the synthesis to trials using contemporary surfactant preparations, catheters, and CPAP thresholds. The window spans five years and seven months rather than five years. Randomised trials published before 2021 that would otherwise have satisfied every eligibility criterion were excluded on publication date alone; these are enumerated with reasons in the full-text exclusion list, and four of them are discussed later for historical context only, without contributing to the synthesis. We regard this restriction as the least defensible methodological decision in the review and have retained it only for transparency with the original protocol-free approach; its consequences are addressed in the Limitations.

Study Selection

All records were exported to a reference manager and de-duplicated. Two reviewers independently screened titles and abstracts, then independently assessed the full texts of all potentially eligible records. Agreement between reviewers was quantified with Cohen's kappa at each stage, and disagreements were resolved by discussion, with a third reviewer adjudicating where consensus could not be reached; the observed agreement statistics and the number of adjudications are reported in the Results. Where multiple publications reported the same trial, the most complete primary report was used as the index publication.

Data Extraction

Data were extracted independently and in duplicate onto a piloted standardised form. Extracted variables comprised first author, year, country and number of centres, trial registration identifier, design and masking, enrolment period, gestational age and birth weight criteria, postnatal age at enrolment, the FiO2 threshold triggering surfactant, mode of non-invasive support, sample size per arm, surfactant preparation and dose, catheter or delivery device, use of premedication or sedation, prespecified intubation criteria, primary and secondary outcomes, effect estimates with measures of precision, and reported adverse events. No extracted variable is listed here without being reported somewhere in the manuscript or supplementary files. Discrepancies were reconciled against the source publication. No study authors were contacted, and no missing data were imputed.

Outcomes

The primary outcome of the review was the need for endotracheal intubation and invasive mechanical ventilation within 72 hours of birth or of the study intervention, as defined by each trial. Secondary outcomes were the composite of death or BPD at 36 weeks' postmenstrual age, BPD alone among survivors, all-cause mortality before discharge, severe (grade III or IV) intraventricular haemorrhage, air leak or pneumothorax, haemodynamically significant patent ductus arteriosus, necrotising enterocolitis, retinopathy of prematurity, need for a repeat surfactant dose, duration of invasive and non-invasive respiratory support, duration of hospitalisation, and procedure-related adverse events.

The review's primary outcome was not the primary outcome of every included trial. It was a secondary outcome in the trials of Sabzehei et al., whose primary endpoint was a set of clinical outcomes after surfactant administration, and of Anand et al., whose primary endpoint was the total duration of respiratory support; and it was captured within a broader composite endpoint of respiratory failure in the trial of Dani et al. Outcomes reported as secondary are more exposed to selective and incomplete reporting than primary endpoints. We flag this explicitly rather than recording those cells only as unreported. Throughout the manuscript, BPD among survivors to 36 weeks and the composite of death or BPD are reported as separate outcomes and are never conflated.

Risk-of-Bias Assessment

Risk of bias was appraised independently by two reviewers using the revised Cochrane risk-of-bias tool for randomised trials (RoB 2) [10], across the five domains: randomisation process (D1), deviations from intended interventions (D2), missing outcome data (D3), measurement of the outcome (D4), and selection of the reported result (D5). Domain-level judgements of low risk, some concerns, or high risk were assigned using the tool's signalling questions, and overall judgements were derived with the recommended algorithm. Assessments were made for the review's primary outcome, and disagreements were resolved by consensus.

Certainty of Evidence

The certainty of the evidence for the primary outcome and for selected secondary outcomes within each of the four comparisons was rated using the GRADE approach [11]. Bodies of evidence from randomised trials began at high certainty and were downgraded for risk of bias, inconsistency, indirectness, imprecision, and suspected publication bias. Ratings and the reasons for each downgrade are presented in the summary of findings table. Because the four comparisons differ substantially in the number of contributing trials, in the risk of bias of those trials, and in the precision of their estimates, GRADE was applied separately within each comparison rather than across the review as a whole.

Assessment of Reporting Bias

Formal statistical assessment of small study effects or publication bias, such as funnel plot asymmetry or Egger's test, was not undertaken because no meta-analysis was performed and because no comparison contained the minimum of 10 trials with usable effect estimates conventionally required for such methods. Reporting bias was therefore considered qualitatively at the outcome level through RoB 2 domain D5, through comparison of published reports against trial registry entries where a registration was available, and by noting where trials reported a statistically significant result narratively without the underlying event counts. The possibility of unassessed publication bias is acknowledged in the Limitations.

Data Synthesis

No meta-analysis was performed, and the review is reported in accordance with the Synthesis Without Meta-analysis (SWiM) reporting guideline [12]. Studies were grouped for synthesis by comparator, giving four comparisons: LISA or MIST against INSURE; MIST against CPAP alone; nebulised surfactant against CPAP alone; and nebulised surfactant against LISA. Within each group, the standardised metric was the risk ratio (RR) with a 95% confidence interval (CI) for the primary outcome.

We considered pooling the eight trials of LISA or MIST against INSURE, which share a population, an intervention, a comparator, and a broadly similar primary endpoint, and we recognise that a random-effects model with a prespecified subgroup analysis by background mode and a formal test for interaction would test this review's central hypothesis directly rather than by inspection. We did not do so for a specific and, in our view, decisive reason: three of the eight trials do not report event counts for the outcome and are therefore not estimable. Those three trials are precisely the ones that determine the interaction, since two used nasal CPAP and reported a benefit or a null result and one used NIPPV. Pooling the five estimable trials, or testing an interaction within them, would have produced a subgroup analysis resting on two trials in one stratum and would have omitted the very evidence that bears on the question. A pooled estimate generated in that way would have conveyed a precision the underlying data do not support.

Instead, and in line with the SWiM guideline, effect direction and precision are presented at the level of the individual trial. Where a trial reported only a p-value or bare percentages, we calculated the RR and its 95% CI from the published two-by-two table using the Katz logarithmic method; these are arithmetic derivations of published data rather than re-analyses, and every such estimate is flagged in the tables and figures as calculated by the review authors. Individual trial estimates for the LISA or MIST against INSURE comparison are displayed as a forest plot deliberately presented without a summary diamond, so that direction and precision can be judged at a glance without implying a pooled result. Synthesis is not conducted by counting statistically significant results: a non-significant finding in a small trial does not establish equivalence or absence of benefit, and the difference in statistical significance between two trials does not itself demonstrate an interaction. Effect estimates reproduced from trial reports are presented as originally published and are not recalculated.

Results

Study Selection

Database and register searching identified 431 records: 379 from databases (PubMed, n = 118; Web of Science, n = 149; Scopus, n = 64; Embase, n = 48) and 52 from ClinicalTrials.gov. After removal of 236 duplicates, 195 records were screened for relevance and 98 were excluded at title and abstract stage. All 97 reports sought for retrieval were obtained and assessed for eligibility, of which 86 were excluded: 38 review articles, editorials, or opinion letters; 27 studies that were not RCTs; and 21 studies that did not meet the remaining inclusion criteria, including publication before 1 January 2021. Eleven RCTs satisfied all eligibility criteria and were included. The selection process is shown in Figure 1.

**Figure 1:**
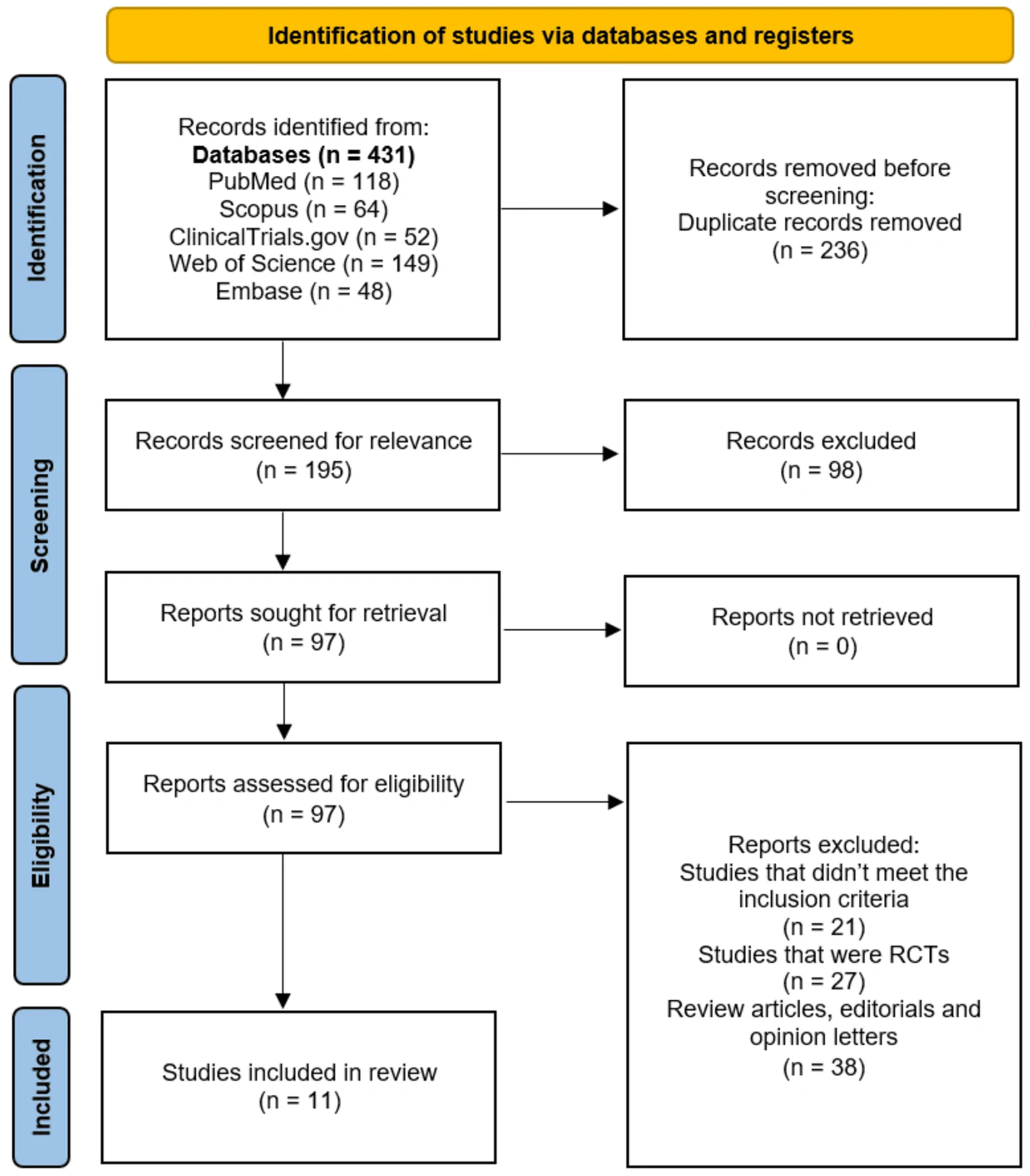
PRISMA 2020 flow diagram of study identification, screening, eligibility assessment, and inclusion PRISMA: Preferred Reporting Items for Systematic Reviews and Meta-Analyses; RCT: randomised controlled trial.

Characteristics of the Included Studies

Results are presented in four comparisons, in the following order: LISA or MIST compared with INSURE; MIST compared with CPAP alone; nebulised surfactant compared with CPAP alone; and nebulised surfactant compared with LISA. A risk-of-bias summary and a GRADE assessment follow.

The 11 included trials enrolled 1,455 preterm infants and were published between 2021 and 2025. Their characteristics are summarised in Table 2 and their technical and procedural details in Table 3, presented in chronological order of publication: the OPTIMIST-A trial [13], Akçay et al. [14], Pareek et al. [15], the CURONEB trial reported by Dani et al. [16], Sabzehei et al. [17], Anand et al. [18], Kaleem et al. [19], Mishra et al. [20], Sayed et al. [21], Hooda et al. [22], and Jiang et al. [23]. Grouped by comparator, eight trials enrolling 792 infants compared LISA or MIST with INSURE [14,15,17-22]; one multinational trial of 485 infants compared MIST with a sham procedure and continuation of CPAP alone [13]; one multinational three-arm trial of 129 infants compared two doses of nebulised poractant alfa plus nasal CPAP with nasal CPAP alone [16]; and one exploratory multicentre trial of 49 infants compared nebulised surfactant delivered by a vibrating mesh nebuliser with LISA [23].

**Table 2:** Characteristics of the 11 included randomised controlled trials Risk ratio and 95% confidence interval calculated by the review authors from the published two-by-two table. Values are for the intervention compared with the comparator. Sample size is total (intervention/comparator). BPD: bronchopulmonary dysplasia; CPAP: continuous positive airway pressure; FiO2: fraction of inspired oxygen; Fr: French gauge; INSURE: intubation-surfactant-extubation; LISA: less invasive surfactant administration; MIST: minimally invasive surfactant therapy; NIPPV: nasal intermittent positive pressure ventilation; OR: odds ratio; PDA: patent ductus arteriosus; RCT: randomised controlled trial; RR: risk ratio; wk: weeks.

Study	Country	Design	Population	Sample size	Intervention	Comparator	Key finding for the review's primary outcome
Dargaville et al., 2021 (OPTIMIST-A) [13]	Multinational, 33 centres	Masked, sham-controlled RCT	25+0-28+6 wk; CPAP; FiO2 ≥0.30 at <6 h	485 (241/244)	MIST, poractant alfa 200 mg/kg	Sham procedure; CPAP continued	Intubation <72 h 36.5% vs 72.1% (RR 0.50, 0.40-0.64). BPD among survivors reduced (RR 0.83, 0.70-0.98); composite of death or BPD not significantly reduced (RR 0.87, 0.74-1.03)
Akçay et al., 2021 [14]	Turkey	Open-label RCT	<34 wk; NIPPV	78 (42/36)	LISA via umbilical catheter	INSURE	No significant difference in intubation <72 h or any morbidity; event counts not reported. pCO2 fell faster after LISA
Pareek et al., 2021 [15]	India	Open-label pilot RCT	28+0-36+6 wk; CPAP or NIPPV	40 (20/20)	LISA, poractant alfa 100 mg/kg	INSURE	Intubation <72 h 6/20 vs 6/20 (RR 1.00, 0.51-1.97)
Dani et al., 2022 (CURONEB) [16]	Multinational (Europe)	Open-label 3-arm RCT	28+0-32+6 wk; CPAP	129 (43 per arm; 42/41/43 analysed)	Nebulised poractant alfa 200 or 400 mg/kg	Nasal CPAP alone	Respiratory failure <72 h 24/42 (57%), 20/41 (49%), 25/43 (58%); RR vs CPAP alone 0.98 (0.68-1.42) and 0.84 (0.56-1.26)
Sabzehei et al., 2022 [17]	Iran	RCT	28-36 wk; CPAP	112 (56/56)	MIST, poractant alfa 200 mg/kg	INSURE	No significant difference in need for mechanical ventilation (a secondary endpoint in this trial); event counts not reported. Shorter stay and less PDA with MIST
Anand et al., 2022 [18]	India	Unblinded RCT	26-34 wk; CPAP, FiO2 >0.30 at <6 h	150 (74/76)	LISA via 8-Fr feeding tube	INSURE	Invasive ventilation reduced (RR 0.50, 0.26-0.96); CPAP failure reduced (RR 0.55, 0.34-0.89). Primary trial endpoint was duration of respiratory support
Kaleem et al., 2023 [19]	Pakistan	RCT	RDS deteriorating on CPAP	72 (36/36)	MIST, poractant alfa 200 mg/kg	INSURE	Invasive ventilation 8/36 vs 17/36 (p = 0.047; RR 0.47, 0.23-0.95). Trial at high risk of bias
Mishra et al., 2023 [20]	India	Open-label RCT	28-36 wk; NIPPV	150 (75/75)	LISA via thin catheter	INSURE	Intubation and ventilation <72 h 30/75 vs 30/75 (RR 1.00, 0.68-1.48)
Sayed et al., 2024 [21]	Egypt	RCT	28-36 wk; CPAP, surfactant <6 h	90 (45/45)	MIST via thin catheter	INSURE	Need for mechanical ventilation significantly reduced; event counts not reported, so not estimable. Trial at high risk of bias
Hooda et al., 2025 [22]	India	Open-label RCT	26-34 wk; NIPPV, surfactant <6 h	100 (50/50)	LISA, bovine surfactant 100 mg/kg	INSURE	Invasive ventilation <72 h 14/50 vs 15/50 (p = 0.86; RR 0.93, 0.51-1.72)
Jiang et al., 2025 [23]	China, seven centres	Open-label exploratory RCT	25+0-32+6 wk; <1500 g; CPAP	49 (25/24)	Nebulised poractant alfa 200 mg/kg (vibrating mesh)	LISA via 6-Fr tube	Ventilation via endotracheal tube <72 h 3/25 vs 5/24 (OR 0.52, 0.11-2.46). Less apnoea and desaturation with nebulisation

**Table 3:** Technical and procedural characteristics of the included trials CPAP: continuous positive airway pressure; FiO2: fraction of inspired oxygen; Fr: French gauge; LISA: less invasive surfactant administration; NIPPV: nasal intermittent positive pressure ventilation; PEEP: positive end-expiratory pressure.

Study	FiO2 threshold for surfactant	Surfactant and dose	Catheter or device	Sedative premedication	Prespecified criteria for intubation
Dargaville et al., 2021 [13]	FiO2 ≥0.30 on CPAP 5-8 cmH2O within 6 h	Poractant alfa 200 mg/kg	16-gauge vascular catheter or LISAcath (Hobart method)	Avoided	Protocolised thresholds applied identically to both arms
Akçay et al., 2021 [14]	FiO2 >0.40	Poractant alfa	Umbilical catheter	Not reported	Not reported
Pareek et al., 2021 [15]	FiO2 0.30-0.40 depending on gestation	Poractant alfa 100 mg/kg	5-Fr infant feeding tube	Avoided	At least two prespecified surfactant criteria within 24 h
Dani et al., 2022 [16]	FiO2 0.25-0.40 on CPAP 5-8 cmH2O	Poractant alfa 200 or 400 mg/kg	Customised vibrating-membrane nebuliser	Not applicable	Prespecified respiratory failure criteria
Sabzehei et al., 2022 [17]	FiO2 >0.40	Poractant alfa 200 mg/kg	5-Fr infant feeding tube	Not reported	Not reported
Anand et al., 2022 [18]	FiO2 >0.30 within 6 h	Surfactant per unit protocol	8-Fr infant feeding tube	Not reported	Prespecified CPAP failure criteria
Kaleem et al., 2023 [19]	FiO2 0.30 with PEEP 6 cmH2O	Poractant alfa 200 mg/kg	Thin catheter	Avoided	Not reported
Mishra et al., 2023 [20]	Per unit protocol on NIPPV	Surfactant per unit protocol	Thin catheter	Not reported	Prespecified intubation criteria
Sayed et al., 2024 [21]	Surfactant criteria within 6 h on CPAP	Surfactant per unit protocol	Thin catheter	Not reported	Not reported
Hooda et al., 2025 [22]	FiO2 ≥0.30 within 6 h on NIPPV	Bovine lipid extract surfactant 100 mg/kg (5 mL/kg)	Thin catheter	Not reported	Prespecified criteria for invasive ventilation
Jiang et al., 2025 [23]	FiO2 >0.25 on CPAP, age 1-12 h	Poractant alfa 200 mg/kg	Vibrating mesh nebuliser (intervention); 6-Fr feeding tube (comparator)	Not reported	Prespecified criteria for endotracheal ventilation

Two trials were multinational or multicentre beyond a single country [13,16]; of the remainder, four were conducted in India [15,18,20,22] and one each in Turkey [14], Iran [17], Pakistan [19], Egypt [21], and China [23]. One trial masked clinicians and outcome assessors through a sham procedure [13]; the other 10 were open-label.

Gestational age windows differed. The most immature population was that of OPTIMIST-A, at 25+0 to 28+6 weeks [13], followed by the vibrating mesh nebuliser trial at 25+0 to 32+6 weeks with birth weight below 1,500 g [23], and the nebulised poractant alfa trial at 28+0 to 32+6 weeks [16]. Among the trials comparing LISA with INSURE, ranges spanned below 34 weeks [14], 26 to 34 weeks [18,19,22], and 28 to 36 weeks [15,17,20,21]. FiO2 thresholds for surfactant ranged from 0.25 [23] and 0.30 [13,18,19,22] to 0.30-0.40 depending on gestation [15] and above 0.40 [14,17]. Poractant alfa 200 mg/kg was the most frequent preparation [13,16,17,19,23]; other trials used poractant alfa 100 mg/kg [15] or a higher-volume bovine lipid extract surfactant at 100 mg/kg [22].

Nasal CPAP was the primary background support in six trials [13,16-19,23] and NIPPV in four [14,20,22], with one trial permitting either CPAP or NIPPV [15]. Catheters included purpose-designed or semi-rigid vascular catheters [13], umbilical catheters [14], and 5-French to 8-French infant feeding tubes [15,17,18,23]. Sedative premedication was avoided in the trials that reported this detail [13,15,19].

LISA or MIST Compared With INSURE

Primary respiratory outcomes are summarised in Table 4, individual trial estimates in Figure 2, and clinical and safety outcomes in Table 5. Of eight trials, three reported a statistically significant reduction in the requirement for invasive ventilation with the less invasive technique; two of these three were judged at high risk of bias [19,21], and this should be borne in mind wherever the finding is cited.

**Table 4:** Primary respiratory outcome (intubation or invasive mechanical ventilation) *Calculated by the review authors from the published two-by-two table using the Katz logarithmic method; all other estimates are as published. CI: confidence interval; other abbreviations as in Table 2.

Study	Comparison (background support)	Intervention	Comparator	Risk ratio (95% CI)	Note
Dargaville et al., 2021 [13]	MIST vs CPAP alone (CPAP)	88/241 (36.5%)	176/244 (72.1%)	0.50 (0.40-0.64); p < 0.001	Published. Trial primary outcome was death or BPD; this was a key secondary outcome
Akçay et al., 2021 [14]	LISA vs INSURE (NIPPV)	Not reported	Not reported	Not estimable	Reported narratively as no significant difference (p > 0.05); event counts not published
Pareek et al., 2021 [15]	LISA vs INSURE (CPAP or NIPPV)	6/20 (30.0%)	6/20 (30.0%)	1.00 (0.51-1.97)	Published; trial primary outcome
Dani et al., 2022 [16]	Nebulised vs CPAP alone (CPAP)	200 mg/kg 24/42 (57%); 400 mg/kg 20/41 (49%)	25/43 (58%)	0.98 (0.68-1.42)*; 0.84 (0.56-1.26)*	Trial primary outcome was a composite of endotracheal surfactant and/or ventilation
Sabzehei et al., 2022 [17]	MIST vs INSURE (CPAP)	Not reported	Not reported	Not estimable	Secondary outcome in this trial; reported narratively as no significant difference
Anand et al., 2022 [18]	LISA vs INSURE (CPAP)	Not reported	Not reported	0.50 (0.26-0.96); p = 0.017	Published estimate; secondary outcome in this trial (primary was duration of support)
Kaleem et al., 2023 [19]	MIST vs INSURE (CPAP)	8/36 (22.2%)	17/36 (47.2%)	0.47 (0.23-0.95)*	Trial reported p = 0.047 only. High risk of bias
Mishra et al., 2023 [20]	LISA vs INSURE (NIPPV)	30/75 (40.0%)	30/75 (40.0%)	1.00 (0.68-1.48)	Published; trial primary outcome
Sayed et al., 2024 [21]	MIST vs INSURE (CPAP)	Not reported	Not reported	Not estimable	Reported narratively as a significant reduction; event counts not published. High risk of bias
Hooda et al., 2025 [22]	LISA vs INSURE (NIPPV)	14/50 (28.0%)	15/50 (30.0%)	0.93 (0.51-1.72)*	Trial reported p = 0.86 only. Trial primary outcome
Jiang et al., 2023 [23]	Nebulised vs LISA (CPAP)	3/25 (12.0%)	5/24 (20.8%)	0.58 (0.15-2.15)*	Trial reported OR: 0.52 (0.11-2.46); p = 0.653. Trial primary outcome

**Figure 2:**
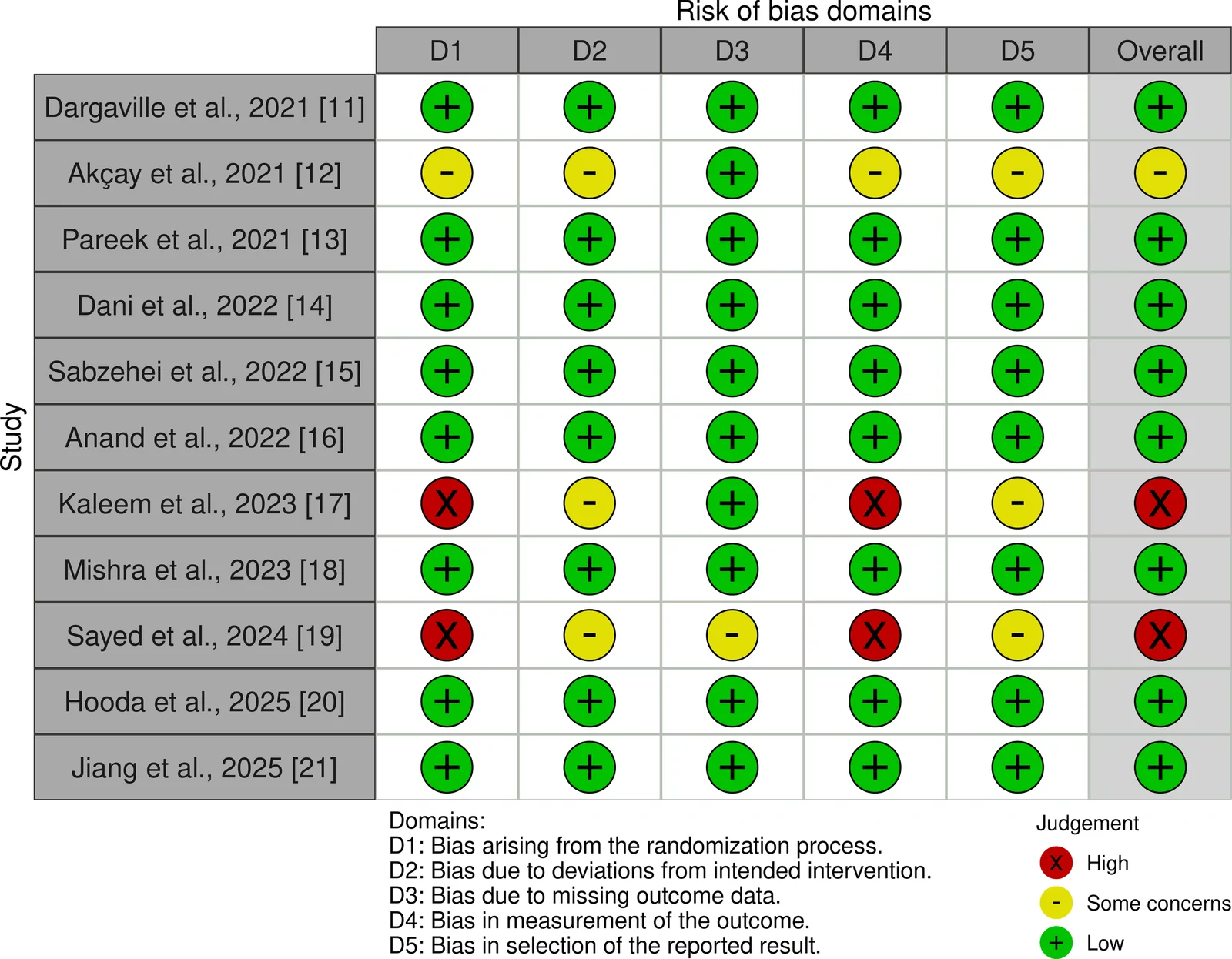
Risk-of-bias traffic-light plot for the 11 included randomised controlled trials, assessed with the revised Cochrane risk-of-bias tool (RoB 2)

**Table 5:** Clinical and safety outcomes Values are interventions compared with a comparator. Entries recorded as "no difference; values not reported" reflect trials that stated an absence of statistical significance without publishing the underlying counts. BPD: bronchopulmonary dysplasia; IVH: intraventricular haemorrhage; OR: odds ratio; PDA/hsPDA: (haemodynamically significant) patent ductus arteriosus; PIVH: periventricular-intraventricular haemorrhage; RR: risk ratio.

Study	BPD (among survivors)	Mortality	Severe IVH	Air leak	PDA	Procedural adverse events
Dargaville et al., 2021 [13]	81/217 (37.3%) vs 102/225 (45.3%); RR 0.83 (0.70-0.98)	Before 36 wk 10.0% vs 7.8%; RR 1.27 (0.63-2.57)	Grade III-IV 7.5% vs 9.8%; RR 0.75 (0.48-1.19)	4.6% vs 10.2%; RR 0.44 (0.25-0.78)	35.3% vs 45.5%; RR 0.77 (0.60-0.99)	Trachea catheterised at first attempt in 76%; positive-pressure inflations in 14%; serious adverse events 10.3% vs 11.1%
Akçay et al., 2021 [14]	No difference; values not reported	No difference; values not reported	No difference; values not reported	No difference; values not reported	No difference; values not reported	No difference reported; pCO2 fell faster after LISA (p < 0.05)
Pareek et al., 2021 [15]	5/20 (25%) vs 2/20 (10%); p = 0.41	3/20 (15%) vs 1/20 (5%); p = 0.60	Any grade 4/20 (20%) vs 2/20 (10%); p = 0.66	Not reported	hsPDA 4/20 (20%) vs 2/20 (10%); p = 0.66	Apnoea or bradycardia 3/20 (15%) in each arm
Dani et al., 2022 [16]	No difference; values not reported	No difference; values not reported	Not reported	No difference; values not reported	Not reported	Well tolerated; no serious adverse events attributed to treatment
Sabzehei et al., 2022 [17]	No difference; values not reported	No difference; values not reported	No difference; values not reported	No difference; values not reported	14.3% vs 30.4%; p = 0.041	Desaturation 19.6% vs 39.3%; p = 0.023
Anand et al., 2022 [18]	No difference; values not reported	No difference; values not reported	No difference; values not reported	No difference; values not reported	No difference; values not reported	Reported; no significant difference
Kaleem et al., 2023 [19]	No difference; values not reported	No difference; discharge more likely after MIST	Numerically lower with MIST; not significant	Pulmonary haemorrhage numerically lower with MIST	Not reported	No significant difference; second dose 2 vs 7 infants (p = 0.075)
Mishra et al., 2023 [20]	No difference; values not reported	No difference; values not reported	No difference; values not reported	No difference; values not reported	No difference; values not reported	9/75 (12%) vs 11/75 (14.6%)
Sayed et al., 2024 [21]	No difference reported	Significantly lower with MIST; values not reported	PIVH significantly lower with MIST; values not reported	No difference reported	Not reported	Reported as well tolerated; values not reported
Hooda et al., 2025 [22]	No difference; BPD or mortality composite also not different	No difference; values not reported	IVH > grade II; no difference	Pulmonary haemorrhage; no difference	hsPDA; no difference	Bradycardia and desaturation; no difference
Jiang et al., 2025 [23]	4/25 (16.0%) vs 6/24 (25.0%); OR 0.57 (0.11-2.34)	1 death in the LISA arm	PIVH; no difference	Pneumothorax; no difference	Not reported	Apnoea 8.0% vs 41.7% (OR 0.10, 0.02-0.54); desaturation <85% 12.0% vs 66.7% (OR 0.07, 0.02-0.30); unmasked bedside ascertainment

Anand et al. randomised 150 infants of 26 to 34 weeks on nasal CPAP to LISA via an 8-French infant feeding tube or to INSURE, and reported that the need for invasive mechanical ventilation was lower after LISA (RR: 0.50, 95% CI: 0.26-0.96; p = 0.017), with a reduction in CPAP failure (RR: 0.55, 95% CI: 0.34-0.89; p = 0.005) [18]. Kaleem et al. randomised 72 neonates deteriorating on nasal CPAP; 8 of 36 infants required invasive ventilation after MIST compared with 17 of 36 after INSURE (p = 0.047; RR calculated by the review authors 0.47, 95% CI: 0.23-0.95), while durations of mechanical ventilation and nasal CPAP did not differ [19]. Sayed et al., in 90 infants of 28 to 36 weeks on CPAP, reported that MIST reduced the need for mechanical ventilation and shortened both mechanical ventilation and CPAP durations but did not publish event counts, so no effect estimate can be derived [21] (Figure 3).

**Figure 3:**
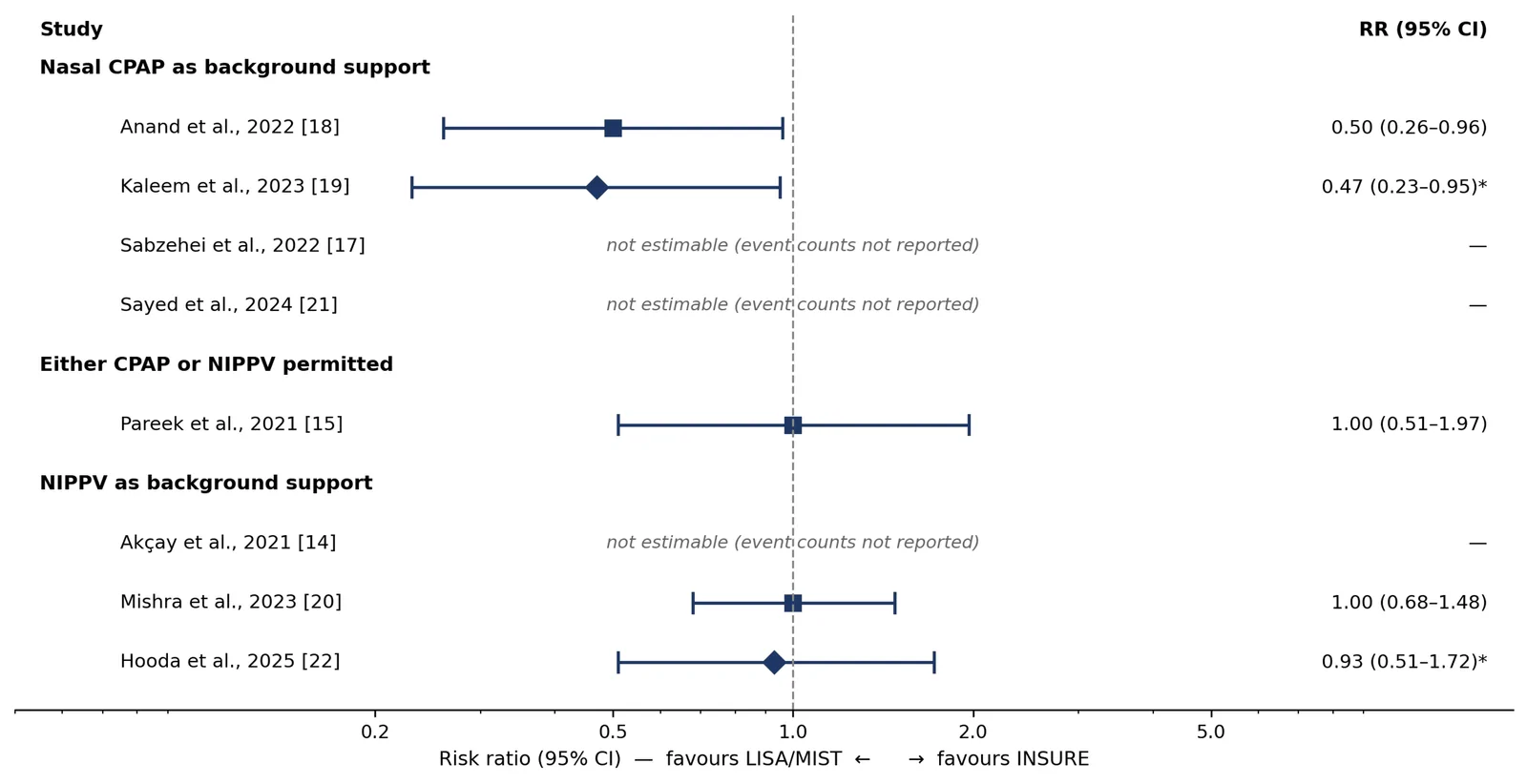
Individual trial estimates for intubation or invasive mechanical ventilation within 72 hours, LISA or MIST compared with INSURE, grouped by background mode of non-invasive support. No summary estimate is shown because no meta-analysis was performed. Squares denote estimates as published; diamonds denote estimates calculated by the review authors from published two-by-two tables (marked *). Three trials reported no event counts and are not estimable. CI: confidence interval; CPAP: continuous positive airway pressure; INSURE: intubation-surfactant-extubation; LISA: less invasive surfactant administration; MIST: minimally invasive surfactant therapy; NIPPV: nasal intermittent positive pressure ventilation; RR: risk ratio.

Five trials reported no significant difference in the primary respiratory outcome. The trials demonstrating no significant reduction in invasive ventilation predominantly used NIPPV as background support, although one trial permitted either CPAP or NIPPV [15] and another used nasal CPAP throughout [17]. Akçay et al. randomised 78 infants below 34 weeks on NIPPV and observed no significant difference in intubation within 72 hours, repeat dosing, duration of NIPPV, or any major morbidity, although the partial pressure of carbon dioxide fell more rapidly after LISA (p < 0.05); event counts were not reported [14]. Pareek et al., in a pilot trial of 40 infants of 28 to 36 weeks supported on either CPAP or NIPPV, reported intubation within 72 hours in 6 of 20 infants in each arm (RR: 1.00, 95% CI: 0.51-1.97) [15]. Mishra et al. randomised 150 infants of 28 to 36 weeks on NIPPV and found intubation and mechanical ventilation within 72 hours in 30 of 75 infants in each arm (RR: 1.00, 95% CI: 0.68-1.48), with procedural adverse events in 12% of the LISA group and 14.6% of the INSURE group [20]. Hooda et al. randomised 100 infants of 26 to 34 weeks on NIPPV to a higher-volume bovine lipid extract surfactant by either technique, with invasive ventilation within 72 hours in 14 of 50 compared with 15 of 50 infants (p = 0.86; RR calculated by the review authors 0.93, 95% CI: 0.51-1.72) and no difference in any secondary outcome [22].

Sabzehei et al. randomised 112 infants of 28 to 36 weeks on nasal CPAP and found no significant difference in the need for mechanical ventilation and the review's primary outcome, which was a secondary endpoint in that trial [17]. The trial's significant findings concerned other secondary outcomes: a shorter hospital stay after MIST (9.19 ± 1.72 compared with 10.21 ± 2.15 days; p = 0.006), a lower incidence of patent ductus arteriosus (14.3% compared with 30.4%; p = 0.041), and less desaturation during the procedure (19.6% compared with 39.3%; p = 0.023). A shorter hospital stay and less patent ductus arteriosus do not establish superiority for the primary respiratory outcome, and this trial is therefore not evidence for the prevention of mechanical ventilation.

Across this comparison, no trial reported a statistically significant excess of death, BPD, severe intraventricular haemorrhage, air leak, necrotising enterocolitis, or retinopathy of prematurity with the less invasive technique. Where differences reached significance, they favoured LISA or MIST, as with the reduction in patent ductus arteriosus reported by Sabzehei et al. [17] and the reductions in periventricular-intraventricular haemorrhage and mortality reported by Sayed et al. [21], the latter in a trial at high risk of bias. Procedure-related adverse events, principally transient bradycardia, desaturation, and surfactant reflux, occurred in 12% to 15% of infants in both arms in the trials that quantified them [15,20].

MIST Compared With CPAP Alone

One trial addressed this comparison. In OPTIMIST-A, 485 infants of 25 to 28 weeks on CPAP at 5 to 8 cmH2O requiring an FiO2 of 0.30 or more within six hours of birth were randomised at 33 units in 11 countries to MIST with 200 mg/kg of poractant alfa, or to a sham procedure with continuation of CPAP [13]. Two distinct outcomes must be separated. The composite of death or physiological BPD at 36 weeks' postmenstrual age occurred in 105 of 241 infants (43.6%) allocated to MIST and 121 of 244 (49.6%) allocated to control, a difference that was not statistically significant (RR: 0.87, 95% CI: 0.74-1.03; risk difference: -6.3%, 95% CI: -14.2% to 1.6%; p = 0.10). Separately, among survivors to 36 weeks, BPD was significantly less frequent after MIST (81 of 217 (37.3%) compared with 102 of 225 (45.3%); RR: 0.83, 95% CI: 0.70-0.98; p = 0.03). Death before 36 weeks was numerically more frequent after MIST (10.0% compared with 7.8%; RR: 1.27, 95% CI: 0.63-2.57; p = 0.51).

MIST halved the need for intubation within 72 hours of birth (88 of 241 (36.5%) compared with 176 of 244 (72.1%); RR: 0.50, 95% CI: 0.40-0.64; p < 0.001) and more than halved pneumothorax requiring drainage (4.6% compared with 10.2%; RR: 0.44, 95% CI: 0.25-0.78; p = 0.005). Other outcomes favouring MIST included intubation at any time (54.8% compared with 81.1%), the requirement for endotracheal surfactant (32.8% compared with 68.4%), patent ductus arteriosus requiring medical therapy (35.3% compared with 45.5%), home oxygen among survivors (14.7% compared with 21.9%), and shorter durations of respiratory support. Serious adverse events were evenly distributed (10.3% compared with 11.1%). An exploratory subgroup analysis identified an interaction between gestational age stratum and allocation for death before 36 weeks, with control favoured at 25 to 26 weeks and MIST favoured at 27 to 28 weeks (p = 0.01 for interaction) [13].

Nebulised Surfactant Compared With CPAP Alone

One trial addressed this comparison. Dani et al. randomised 129 infants of 28+0 to 32+6 weeks with RDS on nasal CPAP at European centres, 43 to each arm, to nebulised poractant alfa 200 mg/kg, nebulised poractant alfa 400 mg/kg delivered through a customised vibrating membrane nebuliser, or nasal CPAP alone; three randomised infants did not receive treatment, giving analysed denominators of 42, 41, and 43 [16]. The primary outcome, respiratory failure within 72 hours defined as the need for endotracheal surfactant and/or mechanical ventilation, occurred in 24 of 42 (57%), 20 of 41 (49%), and 25 of 43 infants (58%) respectively, with no significant differences (RRs calculated by the review authors compared with CPAP alone: 200 mg/kg, 0.98, 95% CI: 0.68-1.42; 400 mg/kg, 0.84, 95% CI: 0.56-1.26). Secondary respiratory outcomes, duration of ventilation, mortality, and BPD did not differ. Nebulised poractant alfa was well tolerated with no serious adverse events attributed to treatment. Enrolment was terminated early following a change in the assessed benefit-risk balance [16].

Nebulised Surfactant Compared With LISA

One trial addressed this comparison directly. Jiang et al. conducted a multicentre, open-label, exploratory randomised trial in seven Chinese neonatal intensive care units in which 49 preterm infants of 25+0 to 32+6 weeks with birth weight below 1,500 g and RDS on nasal CPAP were allocated to nebulised poractant alfa 200 mg/kg by vibrating mesh nebuliser (n = 25) or to LISA via a 6-French feeding tube (n = 24) [23]. Mechanical ventilation via an endotracheal tube within 72 hours occurred in 3 of 25 (12.0%) compared with 5 of 24 infants (20.8%), a difference that was not significant (odds ratio (OR): 0.52, 95% CI: 0.11-2.46; p = 0.653; RR calculated by the review authors 0.58, 95% CI: 0.15-2.15). Blood gas parameters after administration did not differ.

Apnoea of prematurity was recorded in 2 (8.0%) infants in the nebuliser group compared with 10 (41.7%) in the LISA group (OR: 0.10, 95% CI: 0.02-0.54; p = 0.003), and transient desaturation below 85% during administration in 3 (12.0%) compared with 16 infants (66.7%) (OR: 0.07, 95% CI: 0.02-0.30; p < 0.001). Both endpoints were ascertained at the bedside by unmasked staff, and the trial recorded no standardised physiological measures such as heart rate or time to saturation recovery. BPD occurred in 4 (16.0%) compared with 6 (25.0%) infants (OR: 0.57, 95% CI: 0.11-2.34; p = 0.669). There was one death in the LISA group. No significant differences were observed in pneumothorax, periventricular leukomalacia, retinopathy of prematurity, necrotising enterocolitis, periventricular-intraventricular haemorrhage, or need for a repeat dose. The investigators emphasised that the exploratory design and small sample preclude conclusions about comparative efficacy [23].

Synthesis Across Comparisons

A structured summary of findings by comparison is presented in Table 6. In brief, thin-catheter delivery reduced the need for invasive ventilation compared with continuation of CPAP alone in the single trial testing that comparison [13]; findings compared with INSURE were inconsistent in both direction and precision across eight trials, with the three positive results concentrated in trials at high or uncertain risk of bias; and nebulised delivery neither reduced respiratory failure compared with CPAP alone [16] nor differed from LISA in the one small head-to-head trial [23].

**Table 6:** Summary of findings by comparison BPD: bronchopulmonary dysplasia; PIVH: periventricular-intraventricular haemorrhage; RR: risk ratio; other abbreviations as in Table 2.

Comparison	Trials	Infants	Intubation or ventilation	Death and BPD	Safety	Direction of evidence
LISA or MIST vs INSURE [14,15,17-22]	8	792	Reduced in 3 of 8 trials, two of which were at high risk of bias; no difference in 5, all at low risk or with some concerns; 3 trials not estimable	No trial showed harm; one high-risk trial reported lower mortality and PIVH with MIST	Procedural events 12%-15% in both arms where quantified; less desaturation with MIST in 1 trial	Uncertain. Benefit is confounded with trial quality; background mode is a hypothesis, not an established modifier
MIST vs CPAP alone [13]	1	485	Halved: RR 0.50 (0.40-0.64)	BPD among survivors reduced (RR 0.83, 0.70-0.98); separate composite of death or BPD not significantly reduced (RR 0.87, 0.74-1.03)	Pneumothorax reduced (RR 0.44, 0.25-0.78); serious adverse events equal	Favourable for early respiratory outcomes; caution at 25-26 weeks on exploratory subgroup grounds
Nebulised vs CPAP alone [16]	1	129	No difference: 57%, 49%, and 58%	No difference	Well tolerated; enrolment stopped early	Neutral; no demonstrated efficacy at either dose
Nebulised vs LISA [23]	1	49	No difference: RR 0.58 (0.15-2.15)	BPD 16.0% vs 25.0%; not significant	Less apnoea and desaturation with nebulisation, ascertained without masking	Exploratory and underpowered; tolerability signal is hypothesis-generating
All comparisons	11	1,455	Consistent benefit compared with CPAP alone; uncertain compared with INSURE; no difference compared with nebulisation	No technique increased mortality or BPD in any trial	Laryngoscopy-related events dominate the harm profile of thin-catheter techniques	LISA is well supported against no surfactant; its advantage over INSURE is not established

Risk of Bias

Domain-level and overall judgements are presented in Figure 2. Eight trials were judged at low risk of bias across all five domains [13,15-18,20,22,23], reporting adequate sequence generation and allocation concealment, complete or near-complete outcome data, and outcomes consistent with a registered protocol or prespecified analysis plan. OPTIMIST-A was additionally the only trial to achieve effective masking of clinicians and outcome assessors through a sham procedure combined with protocolised intubation thresholds applied identically to both arms [13].

One trial raised some concerns overall [14], recorded for the randomisation process, deviations from intended interventions, measurement of the outcome, and selection of the reported result, reflecting a limited description of sequence generation and allocation concealment, an open-label design with unmasked ascertainment of a partly discretionary endpoint, and the absence of an accessible protocol or prospective registration. Outcome data were complete, and that domain was rated low risk.

Two trials were judged at high risk of bias overall [19,21]. In both, high risk was assigned to the randomisation process and to measurement of the outcome, reflecting inadequate description of sequence generation and allocation concealment together with unmasked ascertainment of the primary respiratory endpoint. Both raised some concerns for deviations from intended interventions and for selection of the reported result, and one raised additional concerns for missing outcome data [21]. These two trials, together with the trial of Sayed et al., account for two of the three positive results in the LISA compared with INSURE.

Certainty of the Evidence

GRADE ratings are presented in Table 7. Certainty was very low for the effect of LISA or MIST compared with INSURE on intubation within 72 hours, downgraded for risk of bias, inconsistency, and imprecision, and low to very low for the secondary outcomes in that comparison. Certainty was high for the reduction in intubation within 72 hours with MIST compared with CPAP alone, and moderate for BPD among survivors, for the composite of death or BPD, and for pneumothorax in that comparison, downgraded for imprecision. Certainty was low for the absence of an effect of nebulised surfactant compared with CPAP alone on respiratory failure, and very low for all outcomes in the comparison of nebulised surfactant with LISA.

**Table 7:** Summary of findings and GRADE certainty of the evidence Certainty was rated separately within each comparison. Evidence from randomised trials begins at high certainty. BPD: bronchopulmonary dysplasia; CI: confidence interval; CPAP: continuous positive airway pressure; GRADE: Grading of Recommendations Assessment, Development and Evaluation; INSURE: intubation-surfactant-extubation; LISA: less invasive surfactant administration; MIST: minimally invasive surfactant therapy; OR: odds ratio; PMA: postmenstrual age; RR: risk ratio.

Comparison	Outcome	Trials (infants)	Effect	Certainty	Reasons for downgrading
LISA or MIST vs INSURE	Intubation or invasive ventilation <72 h	8 (792)	Inconsistent; RRs range 0.47 to 1.00 where estimable; no pooled estimate	Very low	Risk of bias (-1: two trials at high risk, unmasked ascertainment); inconsistency (-1: conflicting direction, not explained by a tested modifier); imprecision (-1: small trials, wide CIs)
LISA or MIST vs INSURE	All-cause mortality before discharge	8 (792)	No trial reported a significant increase; one high-risk trial reported a reduction	Very low	Risk of bias (-1); imprecision (-2: very few events, no trial powered for mortality)
LISA or MIST vs INSURE	BPD among survivors	8 (792)	No significant difference in any trial reporting it	Low	Risk of bias (-1); imprecision (-1)
LISA or MIST vs INSURE	Procedure-related adverse events	8 (792)	12%-15% in both arms where quantified; less desaturation with MIST in one trial	Very low	Risk of bias (-2: unmasked ascertainment of subjective events, incomplete reporting); imprecision (-1)
MIST vs CPAP alone	Intubation <72 h	1 (485)	RR 0.50 (0.40-0.64); 356 fewer per 1,000	High	None. Masked, sham-controlled, protocolised intubation criteria, large and precise effect
MIST vs CPAP alone	Death or BPD at 36 weeks PMA (composite)	1 (485)	RR 0.87 (0.74-1.03); not significant	Moderate	Imprecision (-1: CI includes both no effect and appreciable benefit; recruitment ceased at 81% of target)
MIST vs CPAP alone	BPD among survivors to 36 weeks	1 (485)	RR 0.83 (0.70-0.98)	Moderate	Imprecision (-1: single trial, CI approaches the null)
MIST vs CPAP alone	Pneumothorax requiring drainage	1 (485)	RR 0.44 (0.25-0.78)	Moderate	Imprecision (-1: few events)
MIST vs CPAP alone	Death before 36 weeks PMA	1 (485)	RR 1.27 (0.63-2.57); not significant	Low	Imprecision (-2: few events, CI compatible with substantial benefit or harm)
Nebulised vs CPAP alone	Respiratory failure <72 h	1 (129)	RR 0.98 (0.68-1.42) at 200 mg/kg; 0.84 (0.56-1.26) at 400 mg/kg	Low	Risk of bias (-1: open-label, enrolment stopped early after a change in benefit-risk assessment); imprecision (-1)
Nebulised vs LISA	Ventilation via endotracheal tube <72 h	1 (49)	RR 0.58 (0.15-2.15); not significant	Very low	Risk of bias (-1: open-label); imprecision (-2: 49 infants, CI compatible with large benefit or harm)
Nebulised vs LISA	Apnoea and desaturation during administration	1 (49)	Apnoea OR 0.10 (0.02-0.54); desaturation OR 0.07 (0.02-0.30)	Very low	Risk of bias (-2: subjective endpoints ascertained at the bedside without masking, no standardised physiological measures); imprecision (-1)

Discussion

Principal Findings

This review of 11 randomised trials enrolling 1,455 preterm infants shows that what can be concluded about LISA depends heavily on the comparator. Compared with continuation of CPAP alone at a low oxygen threshold, thin-catheter delivery halved the need for intubation within 72 hours and more than halved pneumothorax requiring drainage, with high certainty for the ventilation outcome [13]. In the same trial, BPD among survivors to 36 weeks was reduced, whereas the separate composite of death or BPD was not; these are different outcomes and the distinction is material to how the trial is cited. Compared with INSURE, the evidence is inconsistent and of very low certainty: three of eight trials reported a reduction in invasive ventilation [18,19,21], five did not [14,15,17,20,22], individual CIs are wide, and three trials cannot be quantified at all because event counts were never published. Compared with nebulised surfactant, the single small head-to-head trial found no difference in ventilation requirements but a markedly higher rate of procedural apnoea and desaturation with thin-catheter delivery [23], while nebulised surfactant itself did not outperform CPAP alone in the only adequately designed trial of that comparison [16].

The Background Mode Hypothesis and Its Confounding With Risk of Bias

The trials showing no advantage of LISA over INSURE predominantly used NIPPV as background support [14,20,22], although one permitted either CPAP or NIPPV [15] and one used nasal CPAP throughout [17], whereas the three trials reporting a reduction in ventilation requirements all used nasal CPAP [18,19,21]. A physiological rationale for such a pattern is available. The benefit of LISA is thought to rest on avoiding a period of positive pressure inflation delivered through an endotracheal tube around the time of surfactant instillation; in immature lambs, a small number of large manual inflations at birth measurably blunts the therapeutic effect of surfactant given subsequently [24]. If the INSURE arm is extubated promptly to NIPPV rather than to CPAP, the intensity and duration of that exposure may be reduced and the differential advantage of avoiding intubation correspondingly attenuated. It is also possible, though to our knowledge untested and therefore offered here as speculation rather than as established mechanism, that the higher mean airway pressure delivered by NIPPV compensates for the pressure loss occurring around a thin catheter during instillation and for transient hypoventilation afterwards.

A rival explanation must be given equal weight, and on the present evidence it is the stronger one. Risk of bias follows almost exactly the same split as background mode. Two of the three trials reporting a benefit, those of Kaleem et al. and Sayed et al., are precisely the two trials this review judges to be at high risk of bias [19,21], while all five trials reporting no difference are at low risk of bias or raise only some concerns [14,15,17,20,22]. Trial quality and background ventilation mode are therefore confounded across this evidence base, and our own risk-of-bias assessment implies that the positive results should be discounted rather than explained. We did not formally test the proposed interaction, and for the reasons set out in the Methods we could not have done so without omitting three of the eight trials. The defensible conclusion is accordingly narrower than the pattern first suggests: the advantage of LISA over INSURE is uncertain in contemporary practice, and background ventilation mode is a hypothesis worth testing prospectively rather than an established effect modifier. A factorial trial randomising both surfactant technique and background mode, or a prospectively planned individual participant data analysis, would settle the question; inspection of published subgroups will not.

Comparison With Existing Evidence

These findings sit in a nuanced relationship with the wider literature. The Cochrane review of surfactant therapy via thin catheter reported reductions in the composite of death or BPD, in the need for mechanical ventilation, and in severe intraventricular haemorrhage [5], and a network meta-analysis ranked thin-catheter administration as the approach most likely to minimise death or BPD among infants on non-invasive support [6]. Registry data from extremely preterm infants below 27 weeks have associated less invasive delivery with improved survival without major morbidity [7]. Our synthesis does not contradict those conclusions, and it is important to be clear that it is not a more reliable estimate of the same quantity: it covers a narrower and more recent window, includes fewer trials, and deliberately offers no pooled estimate. What it adds is the observation that the newer trials, several from low- and middle-income settings and several using NIPPV, do not reproduce the effect seen in the earlier evidence, and that the trials that do reproduce it are the weakest methodologically. Whether that reflects a genuine change in the counterfactual, a difference in populations and thresholds, or simply the play of chance in small trials cannot be resolved here.

The trials of thin-catheter delivery against continuation of CPAP alone address a different clinical question: not how surfactant should be given but whether and when it should be given at all. OPTIMIST-A tested selective surfactant administration at an FiO2 threshold of 0.30, lower than the 0.40 to 0.60 used in the landmark CPAP trials, and its failure to reach significance for the composite primary outcome must be read alongside the fact that recruitment ceased at 81% of target because of the COVID-19 pandemic, leaving the trial underpowered for the hypothesised effect [13]. The confidence interval remained compatible with an absolute reduction of up to 14%. Two earlier randomised trials provide context but were not eligible for this synthesis because they fall outside the publication window and are discussed here for historical purposes only. The AMV trial randomised infants of 26 to 28 weeks to surfactant via thin catheter or standard management and reported reduced mechanical ventilation without a significant effect on BPD [25]. The NINSAPP trial in infants of 23 to 26 weeks similarly demonstrated reduced ventilation exposure with non-intubated surfactant application [26]. The consistent finding across all three is a large reduction in early intubation without a proportionate reduction in chronic lung disease, which reinforces the point made in the Introduction that BPD is multifactorial and unlikely to be substantially modified by a single dose of surfactant on the first day of life.

The exploratory subgroup signal in OPTIMIST-A, in which infants of 25 to 26 weeks allocated to MIST had numerically higher mortality before 36 weeks while those of 27 to 28 weeks did not, requires careful handling [13]. The investigators attributed this partly to a chance imbalance in baseline risk within that stratum, noting that male sex, incomplete antenatal steroid exposure, and multiple birth were each 12% to 14% more frequent in the MIST arm at 25 to 26 weeks. The interaction was not observed for the composite primary outcome or for BPD among survivors, and no deaths were attributed by blinded site investigators to trial participation. It remains a subgroup finding from a trial not powered for it and should be read as a caution rather than a result. It nonetheless argues against extending thin catheter administration to the most immature infants in settings where baseline mortality is high or procedural expertise is still being acquired and supports introducing the technique with structured training, simulation, and audit.

Nebulised surfactant offers the theoretical attraction of eliminating laryngoscopy altogether, but the evidence remains immature. The European trial found no reduction in respiratory failure at either 200 or 400 mg/kg compared with CPAP alone, and enrolment was halted early after reassessment of the benefit-risk balance [16]. Two earlier trials, again outside the publication window and cited here for context only, were more encouraging but methodologically fragile. A blinded randomised trial of nebulised poractant alfa in infants of 29+0 to 33+6 weeks reported a reduction in intubation within 72 hours from 22 of 32 to 11 of 32 infants (RR: 0.53, 95% CI: 0.29-0.95), with the effect confined to the more mature 32+0 to 33+6 week stratum [27]. The largest trial of aerosolised calfactant likewise reported a reduced need for intratracheal bolus surfactant, with the effect concentrated above 31 weeks [28]. The recurring gestational-age dependency is biologically coherent, since low tidal volumes, rapid and variable respiratory rates, small airways, and reduced functional residual capacity all limit aerosol deposition, and the more immature the infant, the greater the surfactant deficit to be overcome. Nebulisation is therefore currently most plausible for moderately and late preterm infants with mild RDS and least plausible for the extremely preterm infants in whom the disease burden is greatest.

The direct comparison of nebulisation with LISA produced the most striking safety contrast in this review, but it will not bear much weight. Apnoea was recorded in 8.0% of infants receiving nebulised surfactant compared with 41.7% of those undergoing LISA, and desaturation below 85% occurred in 12.0% compared with 66.7% [23]. These differences are large, and a physiological explanation is available, since laryngoscopy and catheter placement in an unsedated spontaneously breathing infant provoke vagal and airway reflexes that nebulisation avoids entirely. They rest, however, on 49 infants in an unblinded exploratory trial in which both endpoints were ascertained at the bedside by unmasked staff and no standardised physiological measures were recorded. Transient desaturation during laryngoscopy is an expected accompaniment of the procedure and its clinical consequence is unestablished. An imbalance of this magnitude in a small unblinded trial may reflect differential ascertainment as much as biology, and the finding should be treated as hypothesis-generating. It is nonetheless consistent with longstanding concerns that the procedural burden of LISA has been underappreciated, including prolonged hypoxaemia, bradycardia, raised intracranial pressure, surfactant reflux, and untreated procedural pain [8]. Premedication remains genuinely controversial: sedation improves procedural conditions and comfort but risks respiratory depression that may itself precipitate intubation; national practice differs, and the trials in this review that addressed the issue generally avoided sedation [13,15,19]. The fact that most trials did not report procedural pain or physiological stability in a standardised way is a significant reporting gap.

Implications for Practice and Research

Several implications follow. First, current European consensus guidance recommending LISA as the preferred method of surfactant delivery in spontaneously breathing preterm infants on CPAP [1] is not challenged by this review, which adds no finding that would justify reversing it. What this review does add is that the incremental benefit over INSURE specifically, as opposed to over no surfactant, is less securely established than is often assumed. Second, the expected magnitude of benefit should be conditioned on local practice, and units that already use NIPPV as their default non-invasive mode should not assume the intubation reductions reported in CPAP-based trials while recognising that this caveat is itself provisional. Third, the threshold for administering surfactant may matter as much as the route: OPTIMIST-A showed that intervening at an FiO2 of 0.30 is feasible and reduces intubation, with no discernible difference in treatment effect between infants requiring 0.30 to 0.35 and those requiring more than 0.35 [13]. Fourth, the evidence base is increasingly generated in low- and middle-income settings, and the demonstration that a widely available 8-French infant feeding tube [18] and a higher-volume, more economical bovine lipid extract surfactant [22] can both be used for thin-catheter delivery is of practical value where purpose-designed catheters and porcine surfactant are unaffordable.

Important knowledge gaps remain. No trial in this review enrolled infants below 25 weeks' gestation, so the population with the greatest burden of RDS and BPD is the one with the thinnest evidence. Only one small exploratory trial has compared nebulisation with thin-catheter delivery head-to-head [23], and an adequately powered non-inferiority trial with a prespecified margin is needed before nebulisation can be regarded as a genuine alternative. No included trial reported neurodevelopmental outcomes, respiratory morbidity after discharge, or health economic endpoints, so the durability of the short-term respiratory advantages is unknown. The interaction between surfactant technique and background non-invasive mode has never been tested in a factorial design. Procedural variables likely to modify effectiveness, including catheter type and rigidity, instillation rate and number of aliquots, sedation strategy, operator experience, and the use of lung ultrasound to time administration, have rarely been randomised. Several registered trials identified in ClinicalTrials.gov during screening were ongoing or unpublished at the census date and may alter this picture. Finally, outcome definitions require harmonisation: trials in this review variously defined the primary respiratory endpoint as intubation within 72 hours of birth, within 72 hours of the intervention, or at any time before discharge, and defined BPD physiologically or by oxygen dependence, which materially limits comparability. Reporting also requires improvement, since three of eight trials in the principal comparison published no event counts for the outcome of greatest interest.

Limitations

This review has substantial limitations, several of which bear directly on how its conclusions should be weighted.

It was not prospectively registered, and no protocol was prepared in advance, so readers cannot verify which decisions were prespecified; two important decisions, the publication window and the examination of background ventilation mode, were demonstrably taken after the search. The restriction to publications from 2021 onwards was pragmatic rather than principled, and excluded randomised trials that would otherwise have been eligible and that underpin current guidelines; four such trials are cited in the Discussion for context, which is itself an inconsistency we acknowledge. CENTRAL, the standard register of randomised trials, was not searched, nor were CINAHL or regional indices relevant to the South Asian, Middle Eastern, and North African settings from which most included trials originate, so an eligible trial may have been missed.

Only 1 of the 11 trials was masked, and unmasked ascertainment of a partly discretionary endpoint such as the decision to intubate is an intrinsic threat to validity. Two trials were at high risk of bias, and one raised some concerns; certainty of evidence was very low for the review's principal comparison. Trial quality is confounded with background ventilation mode across that comparison, so the effect modification the review describes could not be disentangled from bias, and it was never formally tested. No meta-analysis was performed, and no pooled estimates are offered; three of eight trials in the principal comparison could not be quantified at all because event counts were not published. No formal assessment of publication bias or small-study effects was possible, so reporting bias across this literature remains unquantified. Three of the four comparisons rest on a single trial each, two of which were small and explicitly exploratory. Finally, no included trial reported long-term neurodevelopmental or respiratory outcomes, and the predominance of single-centre trials means that differences in operator expertise, which strongly influence the success of thin-catheter placement, cannot be recovered from published reports.

## Conclusions

Compared with continuation of CPAP alone at a low oxygen threshold, thin-catheter surfactant delivery reduces early invasive ventilation in infants of 25 to 28 weeks' gestation, halving intubation within 72 hours and reducing pneumothorax requiring drainage. In the same trial, BPD among survivors to 36 weeks was reduced, whereas the separate composite of death or BPD was not. Evidence comparing LISA with INSURE is heterogeneous and of very low certainty: benefit was reported in three of eight trials, two of which were at high risk of bias, and the apparent advantage may be attenuated when NIPPV rather than CPAP is the background mode. That observation is confounded with trial quality in the available evidence and requires prospective testing rather than adoption. Nebulised surfactant remains insufficiently supported: it has not been shown to reduce respiratory failure compared with CPAP alone, and the single head-to-head comparison with LISA was exploratory and underpowered, although it was better tolerated during the procedure itself. Long-term, patient-important outcomes remain uncertain across all four comparisons, since no included trial reported neurodevelopmental or post-discharge respiratory status.

On current evidence, LISA should remain a preferred surfactant delivery strategy for spontaneously breathing preterm infants receiving CPAP when appropriately trained personnel and suitable clinical conditions are available. Priorities for research are adequately powered, masked, or independently adjudicated trials that stratify by background non-invasive mode and formally test for interaction, extension of enrolment below 25 weeks' gestation, a non-inferiority comparison of nebulisation with thin-catheter delivery, and consistent reporting of event counts and neurodevelopmental outcomes beyond neonatal admission.
